# Genome-Wide Analysis of *MBF1* Family Genes in Five Solanaceous Plants and Functional Analysis of *SlER24* in Salt Stress

**DOI:** 10.3390/ijms241813965

**Published:** 2023-09-11

**Authors:** Dongnan Xia, Lulu Guan, Yue Yin, Yixi Wang, Hongyan Shi, Wenyu Li, Dekai Zhang, Ran Song, Tixu Hu, Xiangqiang Zhan

**Affiliations:** 1 State Key Laboratory of Crop Stress Biology for Arid Areas, College of Horticulture, Northwest A&F University, Yangling, Xianyang 712100, China; xiadongnan0110@163.com (D.X.); yinyue2011@nwafu.edu.cn (Y.Y.); wyx0826@nwafu.edu.cn (Y.W.); shihongyan@nwafu.edu.cn (H.S.); lwy15235403214@163.com (W.L.); zdk11@nwafu.edu.cn (D.Z.); songranzk@163.com (R.S.); 2 State Key Laboratory of Crop Stress Biology for Arid Areas, College of Agronomy, Northwest A&F University, Yangling, Xianyang 712100, China; 15737927180@163.com

**Keywords:** Solanaceae species, *MBF1*, gene expression patterns, *SlER24*, salt stress

## Abstract

Multiprotein bridging factor 1 (MBF1) is an ancient family of transcription coactivators that play a crucial role in the response of plants to abiotic stress. In this study, we analyzed the genomic data of five Solanaceae plants and identified a total of 21 *MBF1* genes. The expansion of MBF1a and MBF1b subfamilies was attributed to whole-genome duplication (WGD), and the expansion of the MBF1c subfamily occurred through transposed duplication (TRD). Collinearity analysis within Solanaceae species revealed collinearity between members of the MBF1a and MBF1b subfamilies, whereas the MBF1c subfamily showed relative independence. The gene expression of *SlER24* was induced by sodium chloride (NaCl), polyethylene glycol (PEG), ABA (abscisic acid), and ethrel treatments, with the highest expression observed under NaCl treatment. The overexpression of *SlER24* significantly enhanced the salt tolerance of tomato, and the functional deficiency of *SlER24* decreased the tolerance of tomato to salt stress. *SlER24* enhanced antioxidant enzyme activity to reduce the accumulation of reactive oxygen species (ROS) and alleviated plasma membrane damage under salt stress. *SlER24* upregulated the expression levels of salt stress-related genes to enhance salt tolerance in tomato. In conclusion, this study provides basic information for the study of the *MBF1* family of Solanaceae under abiotic stress, as well as a reference for the study of other plants.

## 1. Introduction

MBF1 is a transcriptional coactivator that is widely distributed in animals, plants, and microorganisms. It plays a critical role in the regulation of gene expression during stress responses [1]. MBF1 consists of two domains: the N-terminal multiprotein bridging factor 1 (MBF1) domain and the C-terminal helix-turn-helix (HTH) domain [1,2]. The HTH domain is essential for maintaining the functional activity of MBF1 [3]. Acting as a transcriptional coactivator, MBF1 facilitates the regulation of gene transcription by bridging the TATA box-binding protein (TBP) to specific transcription factors [4]. Previous studies have demonstrated that MBF1 interacts with various transcription factors, including GCN4 and FTZ-F1 in yeast [5,6], Ad4BP/SF1 in bovines, and ATF1, c-Jun, and c-Fos in humans [7]. MBF1 is involved in both gene transcription and protein translation processes. In yeast, mutations in the *MBF1* gene lead to reduced stability in protein translation [8]. In human embryonic stem cells and HEK-293 cells, MBF1 has been found to bind to mRNA [9], a phenomenon also reported in *Saccharomyces cerevisiae* [10]. Furthermore, MBF1 binds to 30S and 70S ribosomes through a highly conserved HTH-binding domain in Archaea [11].

The MBF1 protein also plays a crucial role in plant resistance to abiotic stress [12,13]. It is primarily located in the nucleus, where it interacts with nuclear proteins to regulate the expression of stress-related genes. For instance, in Arabidopsis, AtMBF1c is normally found in both the cytoplasm and nucleus, but upon exposure to high temperature stress, it rapidly accumulates in the nucleus [13]. In chrysanthemum (*Chrysanthemum morifolium* L.), CmMBF1c interacts with CmHRE2 in the nucleus to regulate downstream genes involved in the waterlogging response, thus enhancing waterlogging tolerance [12]. Additionally, in Arabidopsis, AtSAP5 interacts with AtMBF1c and enhances plant heat tolerance by regulating the expression of two heat shock protein genes [14]. The overexpression of *Hahb-4* from sunflower (*Helianthus annuus*) in Arabidopsis enhances tolerance to drought stress [15], and Hahb-4 interacts with StMBF1 [16]. Notably, MBF1c can also function in the cytoplasm. In wheat (*Triticum aestivum*), TaMBF1c is initially found in both the nucleus and cytoplasm under normal conditions, but upon heat stress, it quickly accumulates in the cytoplasm, where it interacts with RNA-binding proteins to form stress granules, thereby reducing mRNA degradation [17]. Furthermore, MBF1 can directly bind to promoter regions to regulate gene transcription. In Arabidopsis, AtMBF1c binds to CTAGA elements in the promoter regions of 36 heat stress-related genes and regulates the expression of different transcripts, including *AtDREB2A*, two heat shock transcription factors (*HSFs*), and several zinc finger proteins [18]. Similarly, overexpression of the AP2/ERF transcription factor *SlERF.B1* in previous studies increased sensitivity to salt and drought stress, and *SlERF.B1* negatively regulated *SlER24* expression by binding to the promoter [19].

Salinity is one of the major abiotic factors threatening plant production worldwide [20]. Salt stress limits plant growth by increasing the osmotic potential of the soil [21]. The accumulation of ions (Na^+^) in the aboveground parts can reduce the rate of photosynthesis, thereby damaging plant growth [22]. Salt stress can inhibit the growth of plant organs, leading to changes in general plant morphology, such as changes in the root/shoot ratio [23]. In addition, salt stress will cause plants to produce ROS, thus causing oxidative damage to cells and increasing malondialdehyde (MDA) content and ion leakage [24].

Previous studies have confirmed the significant role of the *MBF1* gene in salt stress. For instance, overexpression of *PaMBF1c* from Antarctic moss (*Polytrichastrum alpinum*) in Arabidopsis increased salt tolerance [25], and overexpression of *DgMBF1* in chrysanthemum (*Dendranthema grandiflorum* L.) also enhanced salt tolerance in transgenic plants [26]. Although the function of *MBF1* genes in salt stress has been reported in other plants, there is limited research on Solanaceae plants. In this study, *MBF1* genes were identified from five Solanaceae plants, including tomato (*Solanum lycopersicum* L.), pepper (*Capsicum annuum* L.), eggplant (*Solanum melongena* L.), potato (*Solanum tuberosum* L.), and wolfberry (*Lycium barbarum* L.) [27,28,29,30,31]. A systematic bioinformatics analysis was conducted on the *MBF1* gene family of five Solanaceae plants. The gene expression of the MBF1c subfamily member *SlER24* was induced by treatments with NaCl, PEG, ABA, and ethrel. Reverse genetic approaches were employed to further identify the function of *SlER24* under salt stress. Our results showed that overexpression of *SlER24* enhances salt tolerance. Overall, this study offers valuable insights and a theoretical foundation for the functional investigation of *MBF1* family members in Solanaceae species.

## 2. Results

### 2.1. Identification of MBF1 Genes in Five Solanaceae Species

To identify MBF1 sequences, three AtMBF1 protein sequences (AtMBF1a, AtMBF1a, and AtMBF1c) were used as queries and compared with the protein sequences of five Solanaceae plants—tomato, pepper, eggplant, potato, and wolfberry—using the BLASTP search method and the Hidden Markov Model (HMM) search with the HTH domain file (PF01381). A total of 23 candidate genes were retrieved from five species. To determine the presence of complete HTH domains, the retrieved sequences were compared in the Pfam (Table S1) and CDD databases (Table S2). Two sequences from pepper and wolfberry were found to lack the HTH domain and were removed. Five MBF1 members were identified in tomato and pepper, four in potato and wolfberry, and only three in eggplant (Table 1). The presence of the same Pfam ID indicated that these proteins share a conserved domain. Additionally, when comparing with the MBF1 proteins identified in the TAIR database, all 21 members were annotated as typical MBF1 proteins according to TAIR11 annotation (Table 1). The lengths of genomic DNA (gDNA) and coding sequence (CDS) were 311–28108 bp and 168–2280 bp, respectively. To further elucidate the characteristics of the MBF1 family, the protein molecular weight (MW), isoelectric point (pI), and grand average of hydropathicity (GRAVY) were analyzed (Table S3). The results showed that all 21 MBF1 proteins showed hydrophilicity (ranging from −0.955 to −0.286).

### 2.2. The Classification, Gene Structure, Motif Composition, and Conserved Domain of MBF1 Genes in Five Solanaceae Plants

**Figure 1:**
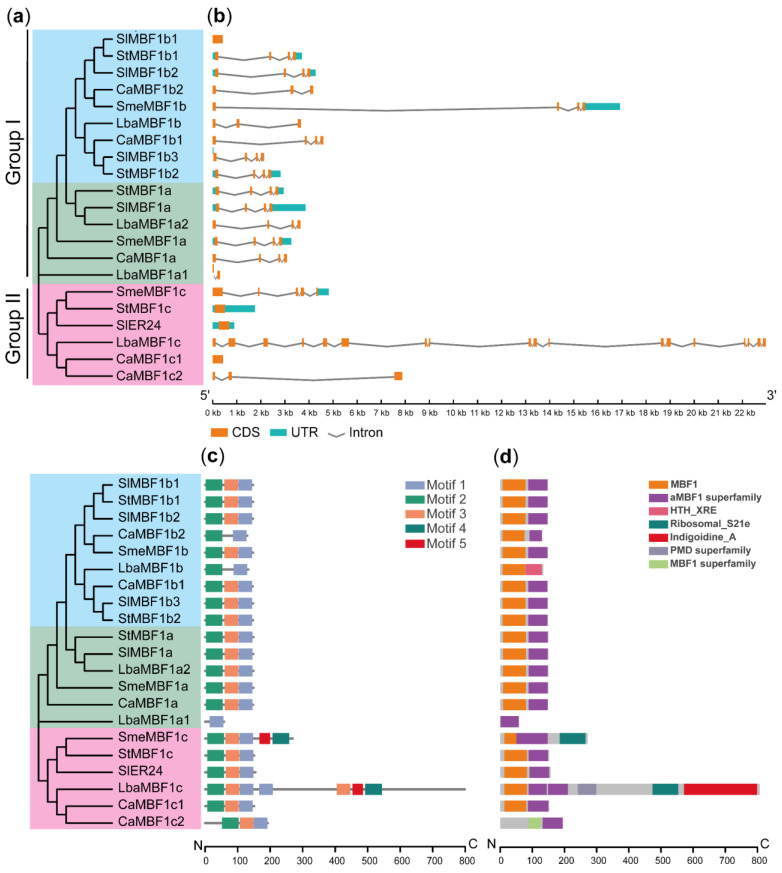
The phylogenetic, gene structure and conserved domain analyses of *MBF1* family from five Solanaceae species. (**a**) Construction of a rootless NJ tree comprising 21 MBF1 protein sequences of five Solanaceae plants. (**b**) The genomic structure of various *MBF1* genes. The light blue box shows UTR regions, the orange box shows exons, and the gray line shows introns. The phylogenetic tree was also constructed using the amino acid sequences of MBF1 proteins in each species. (**c**) The motif distribution of each MBF1 member. (**d**) The conserved protein domain distribution of each MBF1 protein; aMBF1 is the Archaeal ribosome-binding protein aMBF1 domain.

In order to classify the MBF1 family of Solanaceae plants, a phylogenetic tree was constructed using protein sequences. The MBF1 proteins can be classified into two groups: group I includes the MBF1a and MBF1b subfamilies, and group II comprises only the MBF1c subfamily (Figure 1a). The number of exons per gene in Solanaceae plants ranged from 1 to 18, with most *MBF1* genes having 3–5 exons (Figure 1b). Notably, *SlMBF1b1*, *SlER24*, *CaMBF1c1*, and *StMBF1c* had only one exon, *LbaMBF1a1* had two exons, and *LbaMBF1c* had 18 exons.

To further analyze biological functions, motifs and conserved structural domains were identified in 21 MBF1 proteins. Five conserved motifs were identified in the MBF1 protein sequences by MEME software (Figure 1c), and three significant motifs were detected and shown in an additional file (Table S5). Motif 4 and motif 5 are only present in SmeMBF1c and LbaMBF1c. Additionally, the analysis of the protein domains of the 21 MBF1 proteins using the NCBI-Conserved Domain Database (CCD) tool revealed that they are typical members of the MBF1 family (Table S2 and Figure 1c). Interestingly, a Ribosomal_S21e domain was identified in two members of the MBF1c subfamily. Previous studies have confirmed that Archaeal MBF1 binds to 30S and 70S ribosomes through the HTH-binding domain (aMBF1) [11]. Ribosomal_S21e and aMBF1 domains exist simultaneously in the proteins of two MBF1c members (Figure 1d), which may be to allow MBF1 to directly participate in the protein translation process.

### 2.3. Phylogenetic Analysis of the MBF1 Family

**Figure 2:**
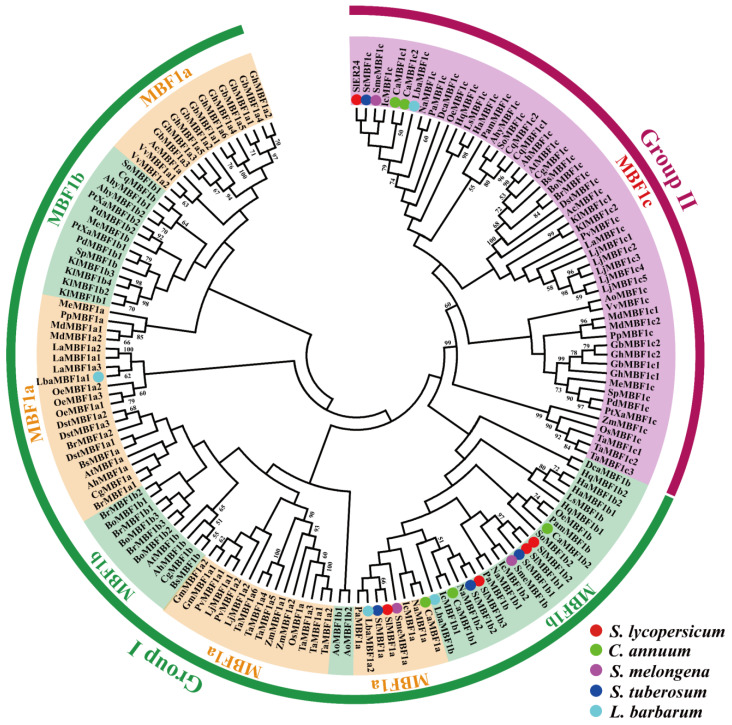
The phylogenetic analysis of the MBF1 family in various plant species. The phylogenetic tree of MBF1 proteins in 43 species. The different colors represent the three different MBF1 subfamilies, and tomato (*S*. *lycopersicum*), pepper (*C*. *annuum*), eggplant (*S*. *melongena*), potato (*S*. *tuberosum*), and wolfberry (*L*. *barbarum*) are marked with red, green, purple, dark blue, and light blue circles, respectively.

A total of 160 amino acid sequences from 43 species (Table S4) were used to construct a phylogenetic evolutionary tree, which illustrates the evolutionary relationships of the MBF1 family (Figure 2). Consistent with previous research findings [1,2], the MBF1 family can be divided into two groups: group I consists of the MBF1a and MBF1b subfamilies, and group II includes only the MBF1c subfamily. It is noteworthy that MBF1a and MBF1b have a closer relationship, suggesting that they may have similar functions. In contrast, MBF1c is a distinct group, indicating its potential involvement in plant stress response and development as an independent role. The fact that MBF1, belonging to the same family of plants, are aggregated on the same branch, particularly in the Solanaceae family, indicates that MBF1 is highly conserved during evolution.

### 2.4. Chromosomal Location, Collinearity, and Gene Duplication Events Analysis of MBF1 Family in Five Solanaceae Plants

**Figure 3:**
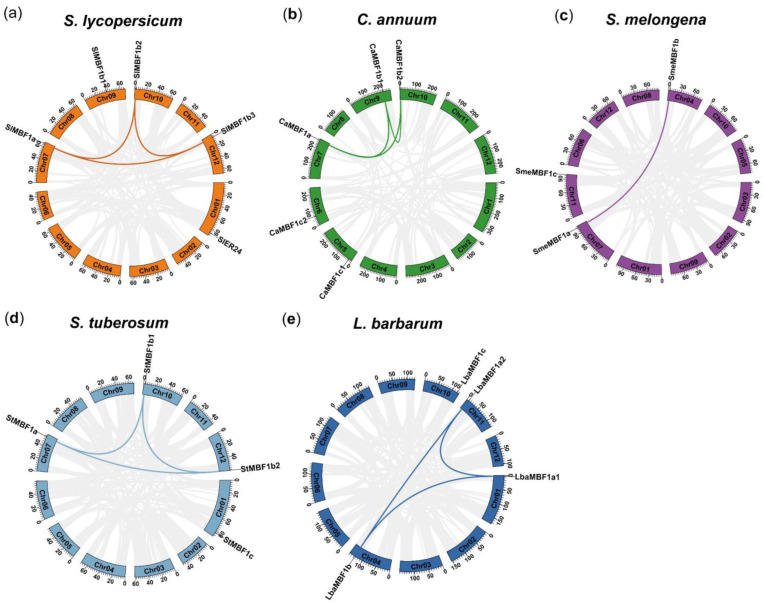
Gene location and collinearity analysis of the *MBF1* family: (**a**) tomato (*S*. *lycopersicum*); (**b**) pepper (*C*. *annuum*); (**c**) eggplant (*S*. *melongena*); (**d**) potato (*S*. *tuberosum*); (**e**) wolfberry (*L*. *barbarum*). The *MBF1* genes in five Solanaceae species are mapped on the different chromosomes. Colored lines join gene pairs with a syntenic relationship.

In this study, the distribution of *MBF1* genes on the chromosomes of five Solanaceae plants was analyzed (Table S9). The results showed that the *MBF1* genes are located on different chromosomes, with most of them located at both ends of the chromosomes (Figure 3). To analyze collinearity, the MCScanX method was used, and collinearity was observed in the genome map. Specifically, collinearity was detected between the MBF1a and MBF1b subfamilies, whereas there was no collinearity between MBF1c and other members (Figure 3).

**Figure 4:**
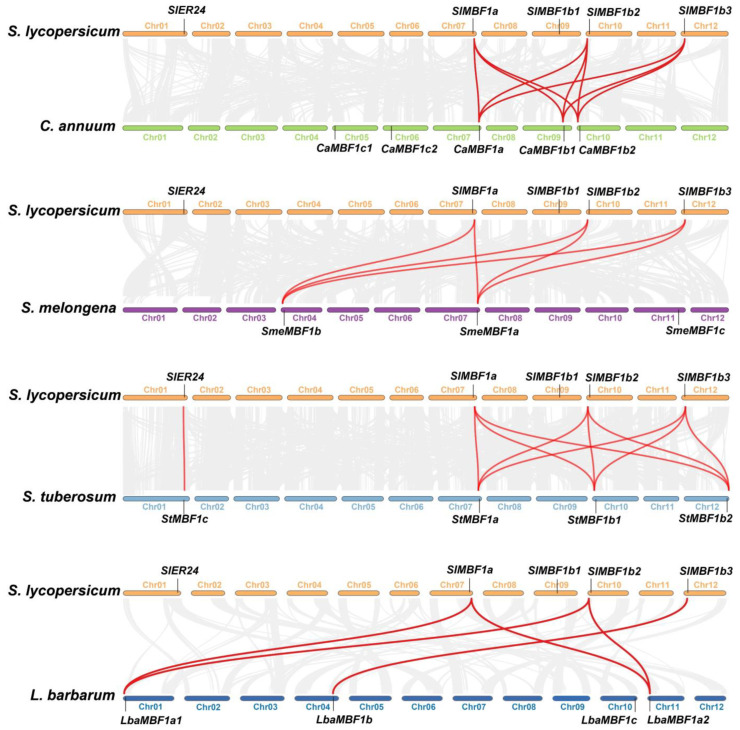
Collinearity analyses of *MBF1* genes between tomato and the four representative species. The gray lines in the background indicate the collinear block with tomato and four other plant species genomes, and the red lines highlight collinearity *MBF1* gene pairs, respectively.

In order to further analyze the evolutionary relationship of the *MBF1* genes in Solanum plants, collinearity analysis was conducted on tomato and other species. The results showed that collinearity was detected between the *MBF1* family of tomato and four other species (Figure 4). The subfamilies MBF1a and MBF1b had collinearity between tomato and all four species, whereas the collinearity of the MBF1c subfamily was only found in tomato and potato. These results indicate that the evolutionary relationship of MBF1a and MBF1b in Solanaceae plants is closer, whereas the evolution of MBF1c is relatively independent.

The formation and expansion of a gene family is closely linked to the process of duplication events. To investigate how *MBF1* evolved, gene duplication events in five Solanaceae plants were analyzed (Table 2), including WGD, tandem duplication (TD), proximal duplication (PD), TRD, and dispersed duplication (DSD). The results indicated that the expansion of the *MBF1* family in five Solanaceae plants was mainly associated with WGD and TRD. The expansion of the MBF1a and MBF1b subfamilies was primarily attributed to WGD, except for *SlMBF1b1*. On the other hand, the expansion of the MBF1c subfamily was mainly associated with TRD. The Ks value was useful in estimating the evolutionary date of WGD events [32]. The collinearity analysis revealed that there were three, three, one, three, and three *MBF1* gene pairs in the genomes of tomato, pepper, eggplant, potato, and wolfberry, respectively (Table 2). The Ks values of *MBF1* gene pairs in the five Solanaceae species ranged from 0.54 to 1.09. The lower Ks values of *MBF1* gene pairs indicated that these genes originated from recent WGD events. Furthermore, the Ka/Ks ratios of duplicate gene pairs were <1 (Table 2), indicating that *MBF1* genes underwent purifying selection during evolution.

### 2.5. Subcellular Localization Analysis of MBF1 Family in Five Solanaceae Plants

Subcellular localization of MBF1 proteins in five different species was predicted using WoLFPSORT. The MBF1a and MBF1b subfamilies were primarily located in the nucleus (Figure S1), and the MBF1c subfamily was distributed in multiple cellular compartments. For instance, CaMBF1c2 was predominantly found in the nucleus, LbaMBF1c was mainly located in the chloroplast, and StMBF1c, SlER24, and CaMBF1c1 were mainly present in the cytoplasm.

### 2.6. Analysis of cis-Elements in MBF1 Family of Five Solanaceae Plants

**Figure 5:**
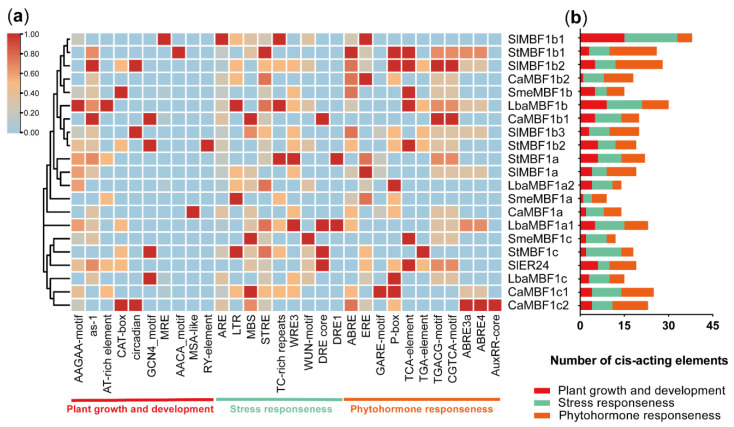
Information on *cis*-acting elements in putative promoter regions of *MBF1* genes from five Solanaceae species. (**a**) The gradient colors in the red grid indicate the number of *cis*-elements in putative promoter regions of *MBF1s*. The data were normalized (ZeroToOne method) using TBtools to construct a heat map. (**b**) The different-colored histogram indicates the *cis*-elements comportment in each category.

To further elucidate the molecular functions and expression patterns of the *MBF1* family, 2000 bp upstream of the start codon (ATG) were extracted from the genomic data of the five species, and these sequences were uploaded to the PlantCARE database for the prediction of *cis*-elements. The results indicated that the promoters of the *MBF1* genes contain a total of 30 *cis*-elements related to plant growth and development, stress response, and plant hormone response, totaling 427 elements (Table S6). The number of each *cis*-element in different *MBF1* promoters was calculated (Table S7), and a heatmap was generated for visualization (Figure 5). In the plant growth and development group (95/427), various regulatory elements were identified, including those involved in endosperm expression (GCN4 motif and AAGAA motif), seed-related *cis*-elements (MSA-like and RY-element), shoot and root meristematic tissue expression (CAT-box), as well as flowering-related elements (AT-rich elements and Circadian). In the stress response group (163/427), various *cis*-elements were identified, including those related to anaerobic induction (ARE), drought induction (MBS, DRE core, and DRE1), low temperature (LTR), stress (TC-rich and STRE), and wounding responsiveness (WRE3 and WUN motifs). Many *cis*-elements (169/427) were classified in the phytohormone responsiveness group, including ethylene (ERE), salicylic acid (TCA element), MeJA (CGTCA-motif and TGACG motifs), Gibberellin acid (TATC-box, GARE-motif, and P-box), auxin (TGA element), and abscisic acid (ABRE, ABRE3a, and ABRE4). These results suggest that the *MBF1* family of Solanaceae plants might be involved in plant development and stress response.

### 2.7. Analysis of the Expression Patterns of SlMBF1s in Tissue and Organ

**Figure 6:**
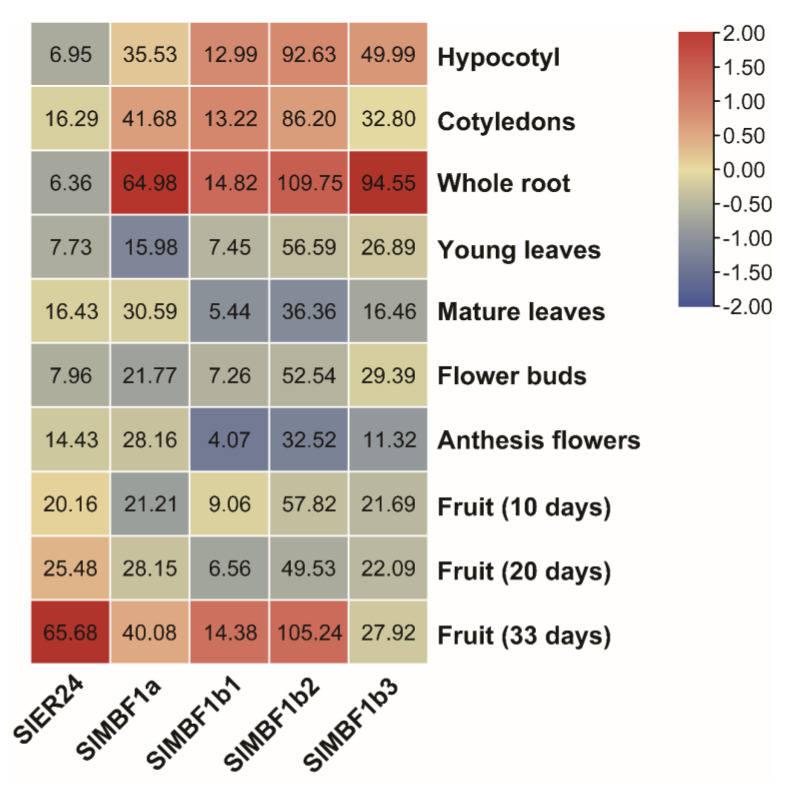
Tissuespecific expression profiles of the tomato *SlMBF1* genes. The expression patterns of the *SlMBF1* genes in the tomato variety LA1589 were investigated using the TFGD database. The data were normalized, and a heatmap was constructed using TBtools, with the expression levels of the gene displayed as numbers in each square.

To explore the expression patterns of the MBF1 family in tomato, expression data of *SlMBF1s* were retrieved from the Tomato Functional Genome Database (TFGD). The expression data of 11 different organs from the tomato variety LA1589 were analyzed to determine the expression patterns of *SlMBF1s* (Figure 6). The results showed that *SlMBF1a*, *SlMBF1b1*, and *SlMBF1b3* exhibited the highest expression levels in the roots compared to other organs. Notably, although *SlMBF1b1* showed expression in various organs, its relative expression level was lower in each organ compared to other members. *SlMBF1b2* displayed higher expression levels in both roots and mature fruits. Furthermore, *SlER24* exhibited the highest expression in mature fruits. These findings indicate that tomato *MBF1s* may participate in the development of different organs. *SlMBF1a*, *SlMBF1b2,* and *SlMBF1b3* may be associated with tomato root development, and *SlMBF1b2* and *SlER24* may be involved in fruit development.

### 2.8. Expression Patterns of SlMBF1s under NaCl, PEG, ABA, and Ethrel Treatments

**Figure 7:**
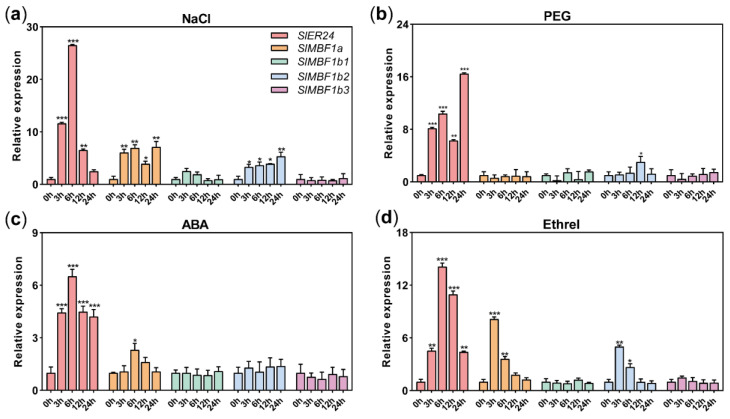
qRT−PCR analysis of the expression patterns associated with *SlMBF1* genes under different stress treatments. (**a**) NaCl treatment, (**b**) PEG treatment, (**c**) ABA treatment, (**d**) ethrel treatment. The 0 h was selected as the control sample to estimate the relative expression in each treatment. Error bars represent SD from three biological replicates. One-way ANOVA was used to analyze significant differences, and asterisks indicate significant differences: * indicates *p* < 0.05; ** indicates *p* < 0.01; *** indicates *p* < 0.001.

The expression patterns of the *SlMBF1s* were analyzed using qRT-PCR under NaCl, PEG, ABA, and ethrel treatments. Under salt stress, *SlER24*, *SlMBF1a*, and *SlMBF1b2* were induced, with *SlER24* reaching its peak expression at 6 h (Figure 7a). After PEG treatment, the expression levels of *SlER24* and *SlMBF1b2* increased (Figure 7b). ABA treatment induced the expression of *SlER24* and *SlMBF1a*, with *SlER24* reaching its maximum expression level at 6 h (Figure 7c). Ethrel treatment induced the expression of *SlER24*, *SlMBF1a,* and *SlMBF1b2*, with *SlER24* reaching its maximum expression level at 6 h (Figure 7d). These results indicate that except for *SlMBF1b1*, *SlMBF1s* can be induced by NaCl, PEG, ABA, and ethrel. It was noteworthy that the expression level of *SlER24* significantly increased under all four treatments, with the highest expression observed under NaCl stress, suggesting that *SlER24* may play a crucial role in the response to salt stress.

### 2.9. Overexpression of SlER24 Increases Salt Tolerance in Tomato

**Figure 8:**
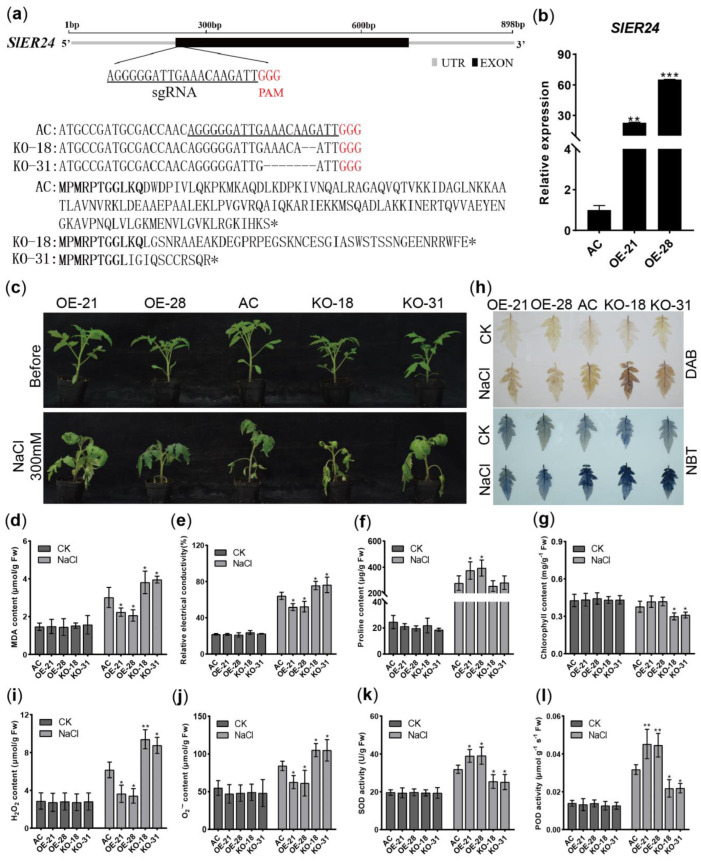
Performance of *SlER24* transgenic tomato plants on salt in soil. (**a**) CRISPR/Cas9-mediated gene-editing types and corresponding amino acid sequences of two *Sler24* homozygous mutants. Underlining presents the sgRNA target sequence, and red font presents protospacer-adjacent motif (PAM) sequences. (**b**) qRT-PCR of *SlER24* in overexpression transgenic plants. (**c**) Phenotypes of 3-week-old AC, *SlER24*-overexpressing plants, and mutant plants after 300 mM NaCl treatment for 5 days. (**d**) MDA content, (**e**) leaf electrolyte leakage, (**f**) proline content, (**g**) chlorophyll content, (**h**) DAB staining and NBT staining, (**i**) H_2_O_2_ content, (**j**) O_2_^−^ content, (**k**) SOD and (**l**) POD activity in leaves from AC, *SlER24*-overexpressing plants and mutant plants under normal and salt stress conditions (300 Mm NaCl for 3 days). Results represent mean values ± SD (*n* = 5). One-way ANOVA analysis using GraphPad software was conducted to determine significant differences, and the average values of the other groups were compared to the average values of the AC group. Asterisks indicate statistically significant differences: * indicates *p* < 0.05; ** indicates *p* < 0.01; *** indicates *p* < 0.001.

Salt stress severely affects the growth and development of tomatoes, and several studies demonstrated the involvement of the MBF1c subfamily in plant responses to abiotic stress [17,33]. To further investigate the impact of *SlER24* on the response to salt stress in tomato, two overexpression transgenic lines (OE-21 and OE-28) with elevated levels of *SlER24* were compared to Ailsa Craig (AC) plants, showing 22-fold and 65-fold increases in *SlER24* transcript levels, respectively (Figure 8b). Additionally, two loss-of-function mutations (KO-18 and KO-31) were generated using the CRISPR/Cas9 system. Two homozygous lines exhibited frameshift mutations, resulting in premature translation termination (Figure 8a). After subjecting three-week-old transgenic plants to 300 mM NaCl treatment, the overexpression lines exhibited significantly stronger tolerance compared to AC and mutant plants (Figure 8c).

Continuing, we measured relative conductivity and MDA content to analyze membrane damage induced by salt stress. Under salt stress, *SlER24*-overexpressing plants exhibited significantly lower MDA content and relative conductivity compared to AC, whereas the mutants displayed higher MDA content and relative conductivity compared to AC (Figure 8d,e), indicating that overexpression plants experience less membrane damage. Conversely, under salt stress, plants overexpressing *SlER24* showed significantly higher proline content than AC, whereas the mutants’ proline content was lower than AC (Figure 8f). Furthermore, after NaCl treatment, the chlorophyll content in the mutant plants was significantly lower than in the overexpression lines and AC (Figure 8g). These findings further support the fact that *SlER24* overexpression lines exhibited enhanced salt tolerance in tomato.

Salt stress leads to rapid accumulation of ROS in plants, and excessive ROS can result in cellular damage [34,35]. To investigate whether *SlER24* affects the accumulation of ROS in tomato under salt stress, 3,30-diaminobenzidine (DAB) and nitroblue tetrazolium (NBT) staining were performed to detect the accumulation of H_2_O_2_ and O_2_^−^, respectively. The results revealed that compared to AC and mutant plants, *SlER24*-overexpressing plants exhibited lower DAB and NBT staining intensities (Figure 8h). Furthermore, H_2_O_2_ and O_2_^−^ levels were significantly lower in *SlER24*-overexpressing plants than in AC, whereas the mutant plants showed significantly higher H_2_O_2_ and O_2_^−^ levels than AC (Figure 8i,j). These findings suggest that overexpression of *SlER24* can reduce ROS accumulation in tomato under salt stress. Additionally, *SlER24*-overexpressing plants exhibited higher activities of superoxide dismutase (SOD) and peroxidase (POD) compared to AC plants, whereas the activities of SOD and POD in mutant plants were significantly lower than in AC under salt stress (Figure 8k,l). In summary, overexpression of *SlER24* can enhance the activity of ROS scavenging enzymes, reducing ROS accumulation and thereby increasing salt tolerance in tomato.

**Figure 9:**
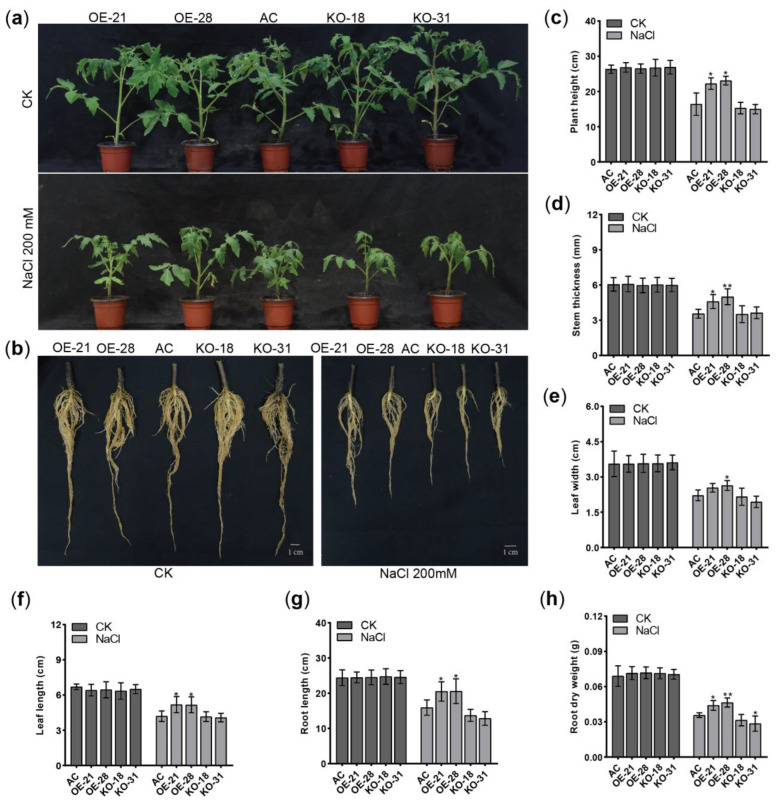
The effect of soil salinity on *SlER24* transgenic tomato plants. (**a**) Phenotypes of 4-week-old AC, *SlER24*-overexpressing plants, and mutant plants after 200 mM NaCl treatment for 14 d. (**b**) Root development status of 3-week-old AC, *SlER24*-overexpressing plants, and mutant plants after 200 mM NaCl treatment for 14 d. (**c**) The height, (**d**) stem thickness, (**e**) leaf width, (**f**) leaf length, (**g**) root length, and (**h**) root dry weight of plants were measured separately. Results represent mean values ± SD (*n* = 6). One-way ANOVA analysis using GraphPad software was conducted to determine significant differences, and the average values of the other groups were compared to the average values of the AC group. Asterisks indicate statistically significant differences: * indicates *p* < 0.05; ** indicates *p* < 0.01.

To investigate the impact of *SlER24* under salt stress on tomato growth and development, three-week-old seedlings were irrigated with 200 mM NaCl and monitored for growth and development over a two-week period. The results showed that *SlER24*-overexpressing plants exhibited improved growth and more vigorous root development compared to AC and mutant plants (Figure 9a,b). Furthermore, the overexpression plants showed significantly greater plant height, stem diameter, leaf width, leaf length, root length, and root dry weight compared to AC (Figure 9c–j). These findings suggest that overexpression of *SlER24* can reduce the damage of salt stress on tomato growth and development. In addition, we examined the effects of *SlER24* under salt stress on tomato seedlings, subjecting them to 200 mM NaCl treatment on 1/2 MS medium. Overexpressing plants showed resistance to NaCl, whereas mutants were more sensitive than AC plants (Figure S2a). In addition, the overexpressing plants showed significantly greater fresh weight, hypocotyl length, and root length compared to AC under salt stress (Figure S2b–d). In conclusion, overexpression of *SlER24* can mitigate the impact of salt stress on tomato growth and development, thereby enhancing the salt tolerance of tomato.

### 2.10. Overexpression of SlER24 Regulates the Expression of Salt Stress-Related Genes

**Figure 10:**
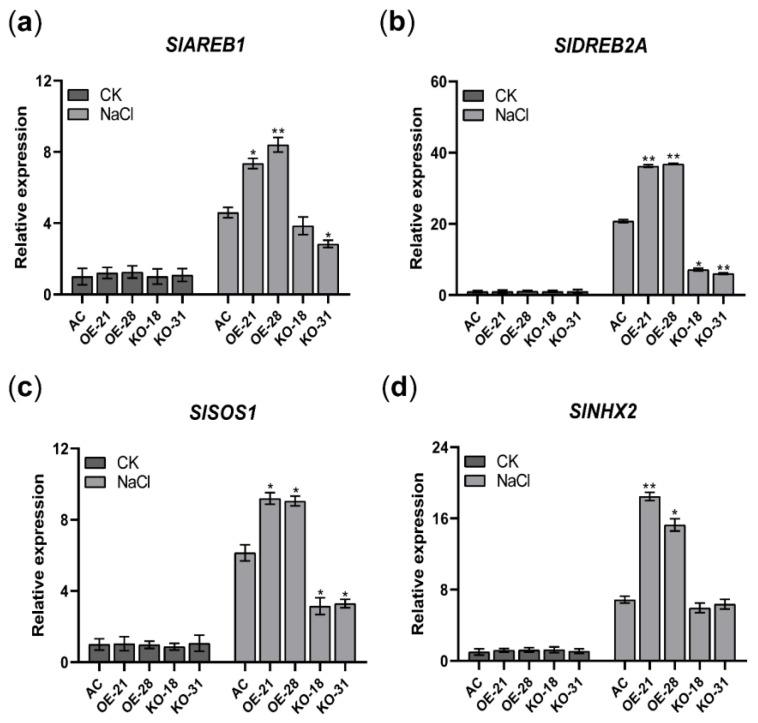
Expression of several stress-responsive genes and phytohormone-related genes in AC, *SlER24*-overexpressing, and mutant plants under normal conditions and salt stress for 6 h. (**a**) Expression of *SlAREB1* gene in AC, *SlER24*-overexpressing, and mutant plants under normal conditions and salt stress for 6 h. (**b**) Expression of *SlDREB2A* gene in AC, *SlER24*-overexpressing, and mutant plants under normal conditions and salt stress for 6 h. (**c**) Expression of *SlSOS1* gene in AC, *SlER24*-overexpressing, and mutant plants under normal conditions and salt stress for 6 h. (**d**) Expression of *SlNHX2* gene in AC, *SlER24*-overexpressing, and mutant plants under normal conditions and salt stress for 6 h. Error bars represent SD from three biological replicates. One-way ANOVA analysis using GraphPad software was conducted to determine significant differences, and the average values of the other groups were compared to the average values of the AC group. Asterisks indicate statistically significant differences: * indicates *p* < 0.05; ** indicates *p* < 0.01.

The above assays indicated that overexpression of *SlER24* positively regulates salt tolerance in tomato. In order to reveal potential molecular mechanisms, qRT-PCR analysis was conducted on selected biologically relevant marker genes associated with salt stress response. The results revealed that there were no significant differences in the expression levels of the salt-related genes *SlAREB1*, *SlDREB2A*, *SlSOS1*, and *SlNHX2* under normal conditions. However, *SlER24*-overexpressing plants showed higher transcription levels of these genes compared to AC, whereas the mutant plants exhibited lower expression levels under salt stress (Figure 10). Overall, these findings demonstrate that *SlER24* positively regulates the transcriptional levels of salt stress-related genes to orchestrate stress response.

## 3. Discussion

MBF1 is a crucial transcriptional coactivator found in animals, plants, and microorganisms, playing a vital role in growth, development, and stress tolerance [1,2,36,37,38,39]. Nevertheless, there is a scarcity of comprehensive information regarding the genome-wide identification and characterization of the *MBF1* gene family in Solanaceous species. In this study, a total of 21 *MBF1* members were identified in five Solanaceae plants and subjected to genome-wide analysis. The MBF1 of plants is divided into group I and group II, further subdivided into three subfamilies: MBF1a, MBF1b, and MBF1c [1]. The MBF1 of five Solanaceae plants is also subdivided into three subfamilies: MBF1a, MBF1b, and MBF1c (Figure 1a). Notably, the protein sequences of two members belonging to the MBF1c subfamily exhibit the presence of a Ribosomal_S21e domain (Figure 1d). Earlier studies have established the interaction of MBF1 protein with ribosomal subunits [11], which are known to enhance the stability of protein translation. Gene families were mainly amplified by five possible methods including WGD, TD, PD, TRD, and DSD [40]. The *MBF1* family of Solanaceae species is mainly expanded in two ways: WGD and TRD. It was discovered that group I (MBF1a and MBF1b) and group II (MBF1c) expand independently through WGD and TRD, respectively (Table 2). Previous studies showed that DSD, WGD, and TRD significantly expanded the *R2R3-MYB* gene family in the five Solanaceae species [41]. The synonymous substitution rate (Ks) represents the background base substitution rate, and Ks values can therefore be used to predict the timing of WGD events [41,42]. The lower Ks values of *MBF1* gene pairs indicate that the expansion of the *MBF1* family in Solanaceae species can be traced back to recent WGD events (Table 2). The Ka/Ks ratio < 1 indicates that the *MBF1* gene evolved under purifying selection. Collinearity analysis was conducted on the *MBF1* genes in tomato, pepper, eggplant, potato, and wolfberry (Figure 3), revealing significant collinearity in the MBF1a and MBF1b subfamilies. Furthermore, the collinearity between Solanaceae species was also examined, and it was observed that the MBF1a and MBF1b subfamilies of tomato displayed clear synteny with other species. However, the collinearity of MBF1c was only evident in tomato and potato (Figure 4).

*MBF1* plays a significant role in growth development and stress tolerance and shows ubiquitous patterns of expression in various organs [1]. There were multiple hormones and abiotic stress-related *cis*-elements in the promoter region of Solanaceae plants (Figure 5), which indicated that the expression of *MBF1* genes was induced by a variety of plant hormones and abiotic stresses. The expression level of *SlER24* gradually increased with fruit maturity and reached its peak during the red ripening stage (Figure 6). Previous studies have confirmed that the *SlER24* gene responds to ethylene [43], which is consistent with the results of this study (Figure 7d). *MBF1* gene expression in plants was regulated by various abiotic stresses [1]. For example, *AtMBF1c* was up-regulated in seedlings after NaCl treatment [44]; high salt, osmotic stress, and heavy metal stress significantly inhibited the expression of *CaMBF1* in chili pepper seedlings [45]; and *VvMBF1* transcript levels increased in response to drought [46]. The gene expression of *SlER24* was induced by NaCl and PEG, and the expression level was higher than the other four members (Figure 7a,b). ABA, as an important stress response hormone, plays an irreplaceable role in salt stress defense [47]. ABA regulation of stomata is an important strategy for plants to cope with NaCl stress [48]. Ethylene accumulates under salt stress, indicating that it plays an important role in salt reactions [49]. After NaCl treatment, the expression levels of *ESE1* and *ERF1*, which are direct targets of *EIN3*, also significantly increase [48]. *EIN2* exhibited salt tolerance by regulating the biosynthesis of ABA and the expression of *RD29B* [48,50]. These results indicate that ethylene regulates plant salt tolerance through crosstalk with ABA. The triple-knockout mutant (abc-) of Arabidopsis *MBF1* genes enhanced seed dormancy and showed hypersensitivity to exogenous ABA [51]. Interestingly, *SlER24* showed a similar expression pattern under NaCl, ABA, and ethrel treatments (Figure 7), suggesting that *SlER24* may play a role in the ABA and ethylene-mediated plant response to NaCl. However, further studies are needed to investigate this possibility.

Previous studies have shown that MBF1c subfamilies are believed to be mainly involved in plant abiotic stress [1]. Several studies have confirmed the significant role of MBF1c in plant abiotic stress, including *AtMBF1c* in Arabidopsis, *TaMBF1c* in wheat, *CmMBF1c* in chrysanthemum, *PaMBF1c* in Antarctic moss, *BocMBF1c* in Chinese kale, and *DgMBF1* in chrysanthemum [12,13,17,25,26,33,52]. However, the specific role of the MBF1c subfamily of Solanaceae plants in salt stress is still unclear. In this study, the biological function of *SlER24* under salt stress was studied, and the results showed that overexpression of *SlER24* enhanced salt tolerance in tomato (Figure 8). Previous studies have confirmed that overexpression of MBF1c member *DgMBF1* enhances the salt tolerance of chrysanthemum [26]. Overexpression of *PaMBF1c* from Antarctic moss in Arabidopsis increased salt tolerance [25]. Overexpression of *SlER24* enhances salt tolerance of tomato, but the role of *SlER24* in other abiotic stresses needs to be further clarified in future research.

Plants enhance resistance through a highly complex and dynamic ROS scavenging system [53,54], and ROS homeostasis and associated antioxidant metabolism are critical for plant survival under salt stress [55]. Antioxidant enzymes, such as POD and SOD, are essential to clear ROS overflow [19]. The overexpression of *VvMBF1* in Arabidopsis reduces the accumulation of ROS (O_2_^−^ and H_2_O_2_) under drought stress and enhances the drought resistance of the plants [46]. Overexpression of *DgMBF1* in chrysanthemum enhances antioxidant enzyme activity and reduces the accumulation of O_2_^−^ and H_2_O_2_ in plants under salt stress [26]. Similarly, overexpression of *CmMBF1c* promotes its ability to scavenge reactive oxygen species and maintain low ROS levels, enhancing its waterlogging tolerance [12]. In this study, the *SlER24*-overexpressing plants showed reduced content of ROS under salt stress, whereas the mutants displayed significantly higher ROS content than that in AC (Figure 8i,j). In addition, the *SlER24*-overexpressing plants exhibited higher activities of antioxidant enzymes, whereas the mutants had lower activities (Figure 8k,l). These findings strongly suggest that *SlER24* overexpression may increase the activities of antioxidant enzymes and reduce the content of ROS, thereby improving the salt tolerance in tomato.

Salinity is one of the major abiotic factors threatening food security worldwide [20]. Salt stress reduces root water absorption by increasing the osmotic potential of the soil, thereby limiting plant growth. The aboveground accumulation of sodium ions (Na^+^) can reduce the rate of photosynthesis, leading to changes in plant morphology, such as a change in the root/shoot ratio [56]. ABA plays a key role in regulating the expression of salt-responsive genes via the ABA-responsive element (ABRE) and the ABRE-binding protein/ABRE-binding factor (AREB/ABF) TFs [57]. Previous studies have confirmed that overexpression of *SlAREB1* can enhance tomato tolerance to salt stress [58,59]. The DREB transcription factor belongs to the AP2/ERF (APETALA2/ethylene-responsive factor) family, which binds to the dehydration response element (DRE) to regulate the expression of a series of downstream genes and enhance plant resistance to abiotic stress [60]. The K^+^/Na^+^ ratio in the cytoplasm is a determining factor for plants to improve Na^+^ tolerance [61]. After plants are subjected to salt stress, a large amount of sodium ions (Na^+^) enters the cells, and potassium ions (K^+^) within the cells undergo efflux, which occurs not only in the roots but also in the leaves [21]. High concentrations of Na^+^ not only alter the osmotic potential of plant cells but also cause toxicity to cells. The Na^+^/H^+^ exchanger SOS1 controls the extrusion and distribution of Na^+^ in tomato plants under salinity conditions [62]. The K/H antiporter (NHX) increases plant salt tolerance by improving K^+^ homeostasis [63]. Similarly, in this study, the expression levels of *SlAREB1*, *SlDREBA2*, *SlSOS1*, and *SlNHX2* genes were higher in *SlER24*-overexpressing plants than those in AC under salt treatment, suggesting that *SlER24* may enhance plant salt tolerance by upregulating the expression of salt stress-related genes.

## 4. Materials and Methods

### 4.1. Identification of MBF1 Genes in Five Solanaceae Species

Three AtMBF1 protein sequences were downloaded from the Arabidopsis database (https://www.arabidopsis.org/, accessed on 5 March 2023) to identify *MBF1* family genes in Solanaceae plants. The genome sequences of tomato, pepper, eggplant, and potato were obtained from the Solanaceae Genomics Network (https://solgenomics.net, accessed on 5 March 2023). The genome sequence and genome annotation files of wolfberry were downloaded from the NCBI database (https://www.ncbi.nlm.nih.gov/, accessed on 5 March 2023), with the accession number PRJNA2. BLASTP searches were performed against native protein databases of Solanaceae species using the three AtMBF1 protein sequences as queries, with E-values < 1 × 10^−5^. HMM profiles of the MBF1 domain (PF08523.13) and HTH domain (PF01381.25) were downloaded from the Pfam database (http://pfam.xfam.org/, accessed on 8 April 2023) [64], and HMMER software (version 3.0) was used to search against protein databases with E-values < 1 × 10^−5^ [65]. Both the Pfam and CD-search Tool (https://www.ncbi.nlm.nih.gov/Structure/bwrpsb/bwrpsb.cgi, accessed on 8 April 2023) databases confirmed the presence of the MBF1 domain and the HTH domain. The *MBF1* genes of Solanaceae species were annotated using the TAIR database and the Pfam database. Additionally, molecular weight (MW), isoelectric point (pI), and hydrophilicity were analyzed using the ExPASy server (https://web.expasy.org/protparam, accessed on 8 April 2023).

### 4.2. The Classification, Gene Structure, Motif Composition, and Conserved Domain of MBF1 Genes in Five Solanaceae Plants

The exon/intron structure of *MBF1* genes was determined using the online program Gene Structure Display Server (http://gsds.gao-lab.org/, accessed on 8 April 2023) [66]. The conserved motifs of each MBF1 protein were identified using MEME_v5.5.2 software (https://meme-suite.org/meme/doc/meme.html, accessed on 8 April 2023) [67]. The domains of each MBF1 protein were obtained from the CDD database (https://www.ncbi.nlm.nih.gov/Structure/cdd/wrpsb.cgi, accessed on 8 April 2023) [68], and the visualization of motifs and conserved domains was performed using TBtools software [69].

### 4.3. Phylogenetic Analysis of the MBF1 Family

The protein sequence data were obtained from Phytozome v13 (https://phytozome-next.jgi.doe.gov/, accessed on 8 April 2023). Protein information for various species is provided in the supplementary file (Table S2). To investigate the phylogenetic relationship of Solanaceae *MBF1* genes, a multiple sequence alignment was performed using MEGA 7.0 software [70]. An alignment-based phylogenetic tree was constructed using the NJ method, with 500 bootstrap replicates conducted for statistical reliability.

### 4.4. Chromosomal Location, Collinearity, and Gene Duplication Events Analysis of MBF1 Family in Five Solanaceae Plants

In this study, the chromosomal location of each *MBF1* gene was collected from the genome data and genome annotation information (Gff3) of five Solanaceae plants. The location of each *MBF1* gene was visualized using TBtools software [69]. MCScanX software was employed to analyze the collinearity of *MBF1* genes [71]. Thresholds were set as follows: E-values < 1 × 10^−5^. To analyze gene duplication events, DupGen_Finder software was utilized.

### 4.5. Subcellular Localization Analysis of MBF1 Family in Five Solanaceae Plants

The subcellular localization of MBF1 proteins was studied using POSRT prediction software (http://psort1.hgc.jp/form.html, accessed on 8 April 2023). Visualization was performed using the heatmap package in the TBtools software [69].

### 4.6. Analysis of cis-Elements in MBF1 Family of Solanaceae

TBtools software was used to extract a 2000 bp sequence upstream of the start codon (ATG) for each *MBF1* gene, which was submitted as the promoter region to the PlantCARE database http://bioinformatics.psb.ugent.be/webtools/plantcare/html/ (accessed on 28 April 2023) for *cis*-element prediction [72]. Visualization was performed using the heatmap package in the TBtools software [69].

### 4.7. Analysis of the Expression Patterns of SlMBF1s in Tissue and Organ

Gene expression data (fragments per kilobase per million reads, FPKM) of *SlMBF1s* were obtained from the Tomato Functional Genomics Database (TFGD, http://ted.bti.cornell.edu/, accessed on 28 April 2023) [73]. The expression data included various tomato organs such as the hypocotyl, cotyledons, whole root, negative meristems, young leaves, mature leaves, young flower buds, anthesis flowers, 10-day post-anthesis fruit, 20-day post-anthesis fruit, and 33-day post-anthesis fruit. Visualization was performed using the heatmap package in the TBtools software [69].

### 4.8. Plant Materials and Treatments for qRT-PCR Analysis

In order to analyze the transcription level of the *SlMBF1s* gene after different stress treatments, one-week-old tomato seedlings were cultured in 1/2 MS solid medium containing 200 mM/L NaCl and 30% polyethylene glycol (PEG, average molecular weight 8000). Whole plants were collected at NaCl and PEG treatments for 0 h, 3 h, 6 h, 12 h, and 24 h, respectively. Four-week-old tomato plants (AC) were sprayed with 100 μM ABA and 100 mg/L ethrel and distilled water (control, CK). The time points for collecting young leaves after treatment with ABA and ethrel were 0 h, 3 h, 6 h, 12 h, and 24 h, respectively. All collected samples were frozen in liquid nitrogen and stored at −80 °C.

Total RNA was extracted with the TRNzol Universal Total RNA Isolation Kit (Tiangen, Beijing, China), according to the manufacturer’s protocol. The samples were removed from storage at −80 °C and frozen in liquid nitrogen before being ground into a powder using a high-throughput homogenizer. One milliliter of TRIzol was added and thoroughly mixed, then allowed to stand at room temperature for 5 min. An amount of 200 µL of chloroform was added and vigorously shaken for 15 s, then allowed to stand at room temperature for 3 min. The mixture was centrifuged at 12,000 rpm at 4 °C for 15 min. The supernatant was transferred to a new centrifuge tube, and an equal volume of isopropanol was added. The tube was mixed by inverting it and allowed to stand for 10 min. After centrifuging at 12,000 rpm, 4 °C, for 10 min, the supernatant was discarded. One milliliter of 75% ethanol wash was added to the precipitate, which was then centrifuged at 10,000 rpm, 4 °C, for 5 min. The ethanol was discarded, and a pipette was used to aspirate the residual ethanol at the bottom of the tube. The sample was air-dried for 3–5 min. Finally, 50 µL of RNase-free double-distilled water (ddH_2_O) was added to promote the complete dissolution of the RNA.

First-strand cDNA was synthesized from 1 µg of total RNA with the HiScript II 1st Strand cDNA Synthesis Kit (Vazyme, Nanjing, China). The following mixture was prepared in an RNase-free centrifuge tube: 5 µL of RNase-free double-distilled water (ddH_2_O), 10 µL of 2× RT Mix, 2 µL of HiScript II Enzyme Mix, 1 µL of Oligo (dT)23VN (50 µM), 1 µL of random hexamers (50 ng/µL), and 1 pg–1 µg of total RNA. The first-strand cDNA synthesis reaction was performed under the following conditions: 5 min at 25 °C, 15 min at 50 °C, and 2 min at 85 °C. The resulting cDNA was stored at −80 °C.

Real-time PCR was performed as described previously [74]. For gene expression detection using real-time fluorescence quantitative PCR, the reaction system and program were as follows: 7.5 μL of 2× qPCR Master Mix, 0.3 μL of forward and reverse primers (10 μM), 0.3 μL of ROX, 1.5 μL of cDNA, and 5.1 μL of ddH_2_O. The reaction program consisted of: initial denaturation at 95 °C for 30 s, denaturation at 95 °C for 10 s, annealing at 60 °C for 30 s, and 40 cycles. All qRT-PCR experiments included three independent biological repetitions. *SlActin2* (Solyc11g005330) was used as a reference gene. The relative gene expression values were calculated using the 2^−∆∆Ct^ method. The information for the Real-time PCR primers was displayed in the supplementary table (Table S8).

### 4.9. Overexpression and CRISPR/Cas9 Vector Construction and Tomato Transformation

The *SlER24* coding region was cloned into the pHellsgate2 vector with *Xho* I and *Xba* I sites driven by the CaMV35S promoter (Figure S3a) [75]. CRISPR/Cas9 vectors were constructed as described by McGrath et al. (Figure S3b) [76]. The designed target sequences were annealed and ligated into the sgRNA-Cas9 vector. The pCAMBIA2301 and sgRNA-Cas9 vectors were double-digested with *Hind* III and *EcoR* I restriction enzymes, followed by ligation using T4 ligase.

### 4.10. Salt Stress Treatments

AC, *SlER24*-overexpressing plants and mutant plants were transferred to pots containing a fixed-weight mixture of soil/perlite/vermiculite (2/1/1, *v*/*v*/*v*) and grown under normal conditions. To investigate the salt stress tolerance, AC and transgenic plants were grown in soil under normal conditions for 3 weeks, after which they were irrigated with 300 mM NaCl solution (3 d intervals).

### 4.11. Chlorophyll, Proline, H_2_O_2_, and O_2_^−^ Content Determination

The total chlorophyll content in leaf tissue was extracted with 1 mL of 95% (*v*/*v*) ethanol as described previously [19]. The sample weighed approximately 0.1 g and was ground in liquid nitrogen, then transferred to a 1.5 mL centrifuge tube. One milliliter of 95% ethanol was added to each centrifuge tube, and the tubes were left in the dark overnight. The tubes were centrifuged at 10,000 rpm for 5 min. An amount of 200 µL of the supernatant was aspirated into an enzyme-labeled plate, and the absorbance of the samples at wavelengths of 649 nm and 665 nm (with 80% acetone as a control) was measured using an enzyme-labeled analyzer. The total chlorophyll content was calculated using the formula: CT = 18.16D_663_ + 6.63D_665_, and the chlorophyll content (mg·g^−1^ FW) = (CT × total volume of extraction solution (mL))/(sample fresh weight (g) × 1000).

Proline was extracted and estimated using the acid ninhydrin method as previously described [74]. Leaf samples of approximately 0.1 g each were taken from salt stress treatments and the control. The samples were ground using liquid nitrogen and transferred to 1.5 mL centrifuge tubes. Then, 1 mL of 3% sulfosalicylic acid was added, and the tubes were extracted in a boiling water bath for 15 min. After cooling, the tubes were centrifuged at 3000 rpm for 10 min, and the filtrate was collected as the proline extraction solution. An amount of 200 μL of the extraction solution was transferred into a new centrifuge tube and mixed with 200 μL of ice acetic acid and 200 μL of acid ninhydrin solution (acid ninhydrin solution: 1.25 g of acid ninhydrin dissolved in 30 mL of ice acetic acid and 20 mL of 2 mol/L phosphoric acid mixture, heated at 70 °C). The mixture was placed in a boiling water bath for 1 h, resulting in a red-colored solution. After cooling, 700 μL of toluene was added to the tube, followed by 30 s of shaking and a brief settling period. The supernatant was transferred to a microplate, using xylene as a blank control, and the absorbance was measured at a wavelength of 520 nm. The concentration of proline was calculated using the equation of the standard curve. The proline content was calculated using the formula: Proline (μg·g^−1^ FW) = (C × V)/(A × W), where V represents the total volume of the extraction solution (mL), C is the concentration of proline in the extraction solution (μg/mL), A is the volume of the extraction solution taken for measurement (mL), and W is the fresh weight of the sample (g).

Hydrogen peroxide (H_2_O_2_) and superoxide (O_2_^−^) in leaves were detected by DAB and NBT, respectively. Hydrogen peroxide (H_2_O_2_) and superoxide (O_2_^−^) in leaves were detected by DAB and NBT, respectively. Detached leaves were vacuum infiltrated with DAB (1 mg·ml^−1^, pH 3.8) or NBT (0.5 mg·ml^−1^, pH 7.8) solution at 28 °C in darkness for 14 h and 2 h, respectively. Before imaging, stained leaves were boiled in 95% ethanol until chlorophyll was removed [19]. The contents of H_2_O_2_ and O_2_^−^ were detected following the method described by Hu et al. [74]. To measure the concentration of O_2_^−^, 0.1 g of leaves were ground with liquid nitrogen in a mortar and transferred to a centrifuge tube. Next, 1 milliliter of chilled phosphate buffer solution (50 mM, pH 7.8) was added, and the homogenate was centrifuged at 5000× *g* for 10 min at 4 °C. The supernatant containing phosphate buffer (pH 7.8) and 10 mM hydroxylammonium chloride was incubated at 25 °C for 20 min. Then, 17 mM p-aminobenzenesulfonic acid and 7 mM α-naphthylamine were added to the mixture. The mixture was further incubated at 25 °C for 20 min and then centrifuged at 1500 g for 5 min. Finally, ethyl ether was added to the mixture. The aqueous phase was used to measure the absorbance at 530 nm. For the measurement of H_2_O_2_ concentration, 0.1 g of leaves were ground with liquid nitrogen in a mortar and transferred to a centrifuge tube. Subsequently, 1 mL of chilled phosphate buffer solution (50 mM, pH 6.8) was added. After centrifugation at 6000× *g* for 15 min, 3 mL of the supernatant and 1 milliliter of 1% titanium sulfate in 20% (*v*/*v*) H_2_SO_4_ were added to a new tube, mixed, and then centrifuged again. The absorbance was measured at 410 nm.

### 4.12. Determination of Antioxidant Enzyme Activities

A total of 100 mg of the leaf sample was ground with 1 mL of ice-precooled 100 mM phosphate buffer (pH 7.0) containing 1 mM EDTA, 0.1% (*v*/*v*) Triton X-100, and 1% (*w*/*v*) PVP40. The homogenate was centrifuged at 12,000 g at 4 °C for 20 min. The resulting supernatant was utilized for determining the activity of antioxidant enzymes, namely, SOD (superoxide dismutase) and POD (peroxidase) were measured as previously described [74].

## 5. Conclusions

This study performed a genome-wide identification and bioinformatics analysis of the *MBF1* gene in five Solanaceae plant species. Based on the bioinformatics and qPCR analysis results, we further investigated the functional role of the MBF1c subfamily member *SlER24* in salt stress response. The overexpression of *SlER24* significantly enhanced the salt tolerance of tomato, and the functional deficiency of *Sler24* decreased the tolerance of tomato to salt stress. *SlER24* reduced ROS accumulation by increasing antioxidant enzyme activity and reduced membrane damage under salt stress. *SlER24* upregulated the expression levels of salt stress-related genes to enhance salt tolerance in tomato. These findings provide novel insights into the function of *SlER24* and contribute to improving plant tolerance to abiotic stress through genetic manipulation. However, the molecular regulatory mechanism of *SlER24* under abiotic stress in tomato remains to be elucidated, and this will be the focus of our future research.

## Figures and Tables

**Table 1:** Summary of MBF1 family annotation information.

Gene ID	Gene Name	Pfam ID	Description	TAIR BlastX
Solyc01g104740.3.1	*SlER24*	PF08523.13/PF01381.25	MBF1/HTH_3	*AtMBF1c*
Solyc07g062400.3.1	*SlMBF1a*	PF08523.13/PF01381.25	MBF1/HTH_3	*AtMBF1b*
Solyc09g055470.1.1	*SlMBF1b1*	PF08523.13/PF01381.25	MBF1/HTH_3	*AtMBF1b*
Solyc10g007350.4.1	*SlMBF1b2*	PF08523.13/PF01381.25	MBF1/HTH_3	*AtMBF1b*
Solyc12g014290.2.1	*SlMBF1b3*	PF08523.13/PF01381.25	MBF1/HTH_3	*AtMBF1b*
CaDEM05G05830	*CaMBF1c1*	PF08523.13/PF01381.25	MBF1/HTH_3	*AtMBF1c*
CaDEM06G07520	*CaMBF1c2*	PF08523.13/PF01381.25	MBF1/HTH_3	*AtMBF1c*
CaDEM07G25960	*CaMBF1a*	PF08523.13/PF01381.25	MBF1/HTH_3	*AtMBF1a*
CaDEM09G17680	*CaMBF1b1*	PF08523.13/PF01381.25	MBF1/HTH_3	*AtMBF1b*
CaDEM10G01710	*CaMBF1b2*	PF08523.13/PF01381.25	MBF1/HTH_3	*AtMBF1b*
Smechr0400261.1	*SmeMBF1b*	PF08523.13/PF01381.25	MBF1/HTH_3	*AtMBF1b*
Smechr0702353.1	*SmeMBF1a*	PF08523.13/PF01381.25	MBF1/HTH_3	*AtMBF1b*
Smechr1102702.1	*SmeMBF1c*	PF08523.13/PF01381.25/PF01249.21	MBF1/HTH_3/Ribosomal_S21e	*AtMBF1c*
Soltu.DM.01G043930.1	*StMBF1c*	PF08523.13/PF01381.25	MBF1/HTH_3	*AtMBF1c*
Soltu.DM.07G023530.1	*StMBF1a*	PF08523.13/PF01381.25	MBF1/HTH_3	*AtMBF1b*
Soltu.DM.10G002940.1	*StMBF1b1*	PF08523.13/PF01381.25	MBF1/HTH_3	*AtMBF1b*
Soltu.DM.12G030120.1	*StMBF1b2*	PF08523.13/PF01381.25	MBF1/HTH_3	*AtMBF1b*
Lba01g00195	*LbaMBF1a1*	PF01381.25	HTH_3	*AtMBF1c*
Lba04g02218	*LbaMBF1b*	PF08523.13/PF01381.25	MBF1/HTH_3	*AtMBF1b*
Lba10g02214	*LbaMBF1c*	PF08523.13/PF01381.25/PF01249.21/PF04227.15	MBF1/HTH_3/Ribosomal_S21e/Indigoidine_A	*AtMBF1c*
Lba11g00477	*LbaMBF1a2*	PF08523.13/PF01381.25	MBF1/HTH_3	*AtMBF1b*

Note: Gene ID is the ID in the genome database; TAIR BlastX is the result obtained by comparing the TAIR database using the BlastX method.

**Table 2:** Expansion mode and ka/ks ratio of *MBF1* gene family.

Gene ID	Gene Name	GDE	Ka	Ks	Ka/Ks
Solyc01g104740.3.1	*SlER24*	TRD			
Solyc07g062400.3.1	*SlMBF1a*	WGD	0.04	0.77	0.05
Solyc09g055470.1.1	*SlMBF1b1*	TRD			
Solyc10g007350.4.1	*SlMBF1b2*	WGD	0.04	1.09	0.04
Solyc12g014290.2.1	*SlMBF1b3*	WGD	0.03	0.88	0.03
CaDEM05G05830	*CaMBF1c1*	TRD			
CaDEM06G07520	*CaMBF1c2*	TRD			
CaDEM07G25960	*CaMBF1a*	WGD	0.05	0.78	0.07
CaDEM09G17680	*CaMBF1b1*	WGD	0.10	0.97	0.10
CaDEM10G01710	*CaMBF1b2*	WGD	0.11	1.03	0.11
Smechr0400261.1	*SmeMBF1b*	WGD	0.05	0.70	0.08
Smechr0702353.1	*SmeMBF1a*	WGD			
Smechr1102702.1	*SmeMBF1c*	TRD			
Soltu.DM.01G043930.1	*StMBF1c*	TRD			
Soltu.DM.07G023530.1	*StMBF1a*	WGD	0.04	0.61	0.07
Soltu.DM.10G002940.1	*StMBF1b1*	WGD	0.04	0.99	0.04
Soltu.DM.12G030120.1	*StMBF1b2*	WGD	0.04	0.71	0.05
Lba01g00195	*LbaMBF1a1*	WGD	0.04	0.92	0.04
Lba04g02218	*LbaMBF1b*	WGD	0.06	0.99	0.06
Lba10g02214	*LbaMBF1c*	TRD			
Lba11g00477	*LbaMBF1a2*	WGD	0.02	0.54	0.04

Note: GDE, gene duplication events; TRD, transposed duplication; WGD, whole-genome duplication. Ka, non-synonymous substitution rate; ks, synonymous substitution rate; ka/ks, ratio of ka to ks.

## Data Availability

The data presented in this study are available in supplementary material.

## Supplementary Materials

The following supporting information can be downloaded at: https://www.mdpi.com/article/10.3390/ijms241813965/s1.

## Abbreviations

qRT-PCR	Quantitative reverse transcription polymerase chain reaction
WGD	Whole-genome duplication
TD	Tandem duplication
PD	Proximal duplication
TRD	Transposed duplication
DSD	Dispersed duplication
FPKM	Fragments per kilobase of transcript per million fragments mapped
MW	Molecular weight
pI	Isoelectric point
HMM	Hidden Markov Model
MS	Murashige and Skoog medium
NCBI	National Center for Biotechnology Information
NJ	Neighbor-joining
